# Lateral Periodontal Cyst – A diagnostic dilemma: Report of a rare case with CBCT and histological findings

**DOI:** 10.1016/j.ijscr.2020.09.089

**Published:** 2020-09-17

**Authors:** Roshni Ramesh, Arun Sadasivan

**Affiliations:** a Department of Periodontics, Government Dental College, Thrissur, Kerala, India; b Department of Periodontics, Sree Mookambika Institute of Dental Sciences, Kulashekaram, Tamil Nadu, India

**Keywords:** Lateral periodontal cyst, Developmental cyst, Endodontically treated tooth, Gingivectomy, CBCT, Case report

## Abstract

**Introduction:**

Lateral Periodontal Cyst (LPC) is considered as a rare developmental odontogenic cyst. It is often diagnosed as an incidental radiographic finding, presenting as a circumscribed round radiolucent area between the roots of vital teeth. LPC usually does not present any clinical features. Differentiating the origin of the lesion from an endodontic or periodontal perspective presents as clinical challenge.

**Presentation of case:**

A female patient presented with an asymptomatic gingival swelling in the lingual aspect of mandibular anterior region. The associated tooth (#34) was endodontically treated 3 years back. A periapical radiograph showed a well-defined round radiolucency on the tooth. Cone beam computed tomography (CBCT) revealed extensive bone destruction. The lesion was surgically excised and histological examination confirmed the diagnosis of LPC. The site healed satisfactorily post-operatively. The case was followed up for a year without any recurrence seen.

**Discussion:**

LPC is a very rare clinical entity, the diagnosis of which requires a detailed case history taking, clinical and radiographic examination are essential to get proper assessment of the pathology. It is said to originate from either the remnants of dental lamina, reduced enamel epithelium or rests of Malassez. LPC presents with a typical histological picture which ensures the confirmatory diagnosis. Surgical enucleation with thorough curettage is the treatment of choice.

**Conclusion:**

By reporting this rare case, we would like to stress to clinicians that there are a wide range of cysts and anatomic structures are present in the canine-premolar region of mandible of which LPC is a rare possibility.

## Highlights

• Lateral periodontal cyst [LPC] is a rare developmental odontogenic cyst.
• It is difficult to differentiate a LPC from a cyst of endodontic origin by clinical and radiographic methods. Histopathological evaluation is the only method to confirm diagnosis.
• Usually seen in association of a vital tooth, but in this case it was seen in a non-vital tooth.
• LPC is usually seen within bone, but in this case besides Intrabony component there was a gingival presentation as a swelling.

## 1 Introduction

The lateral periodontal cyst (LPC) is a non-inflammatory, intra-osseous cyst that arises in close proximity of the roots of vital teeth. It is a relatively rare odontogenic lesion, accounting for just 0.4% of all odontogenic cysts [[1], [2], [3], [4]]. LPC has been classified as an odontogenic cyst of developmental origin according to the World Health Organization (WHO) classification of odontogenic tumours and cysts (4th Edition, 2017) [5]. LPC is usually symptomless and is found while taking radiographs and occurs most frequently in the alveolar bone of the mandibular canine and premolar regions, followed by the anterior segment of maxillary alveolar process [[6], [7], [8]]. In some patients, LPC may clinically present as an asymptomatic gingival swelling in the facial or lingual aspect between two teeth [6,9]. The involved teeth are usually vital, unless they have been affected by periodontitis or dental caries. Also infrequently reported in literature is pain, tenderness and cortical expansion [9]. The typical radiographic picture of LPC is a well circumscribed ovoid or round radiolucent area with a sclerotic margin, usually of diameter less than 1 cm [10]. It usually affects individuals between the fifth and seventh decade of life. No specific racial predilection or sex distribution has been reported in literature [11]. Reports from the literature indicate a possible origin of LPC from either the remnants of dental lamina, reduced enamel epithelium or rests of Malassez [3,6].

From a clinical point of view, it is important to correctly diagnose LPC from a number of other lesions which present similar clinical or radiographic picture. The list includes gingival cyst, odontogenic keratocyst, lateral radicular cyst, glandular odontogenic cysts, lesions of endodontic and periodontal origin [6,9,12]. A correct diagnosis is possible only by histo-pathologic examination (HPE) which will decide the line of treatment to be undertaken. HPE picture of LPC is unique with a distinct type of developmental cyst which has typical epithelial and connective tissue features [2,4]. The treatment is usually with conservative surgical enucleation of the lesion.

We present a case of LPC which located in the canine-premolar region of mandible which occurred in an endodontically treated tooth which has not been reported often. The use of Cone beam computed tomography (CBCT) to assist in the diagnosis and treatment planning is also discussed. A brief literature review of the clinical, radiographic and histological features of LPC is also presented.

This case has been reported in accordance to the SCARE criteria [13].

## 2 Presentation of case

A 49 year-old female patient reported to a private practice at Trivandrum, with a complaint of painless swelling in the lingual aspect of left mandibular canine [#33] and first premolar [#34] region of three months duration. The patient did endodontic treatment of #34 for deep caries three years previously and a crown had been placed. The patient reported no systemic comorbidities, was in good general health.

**Fig. 1:**
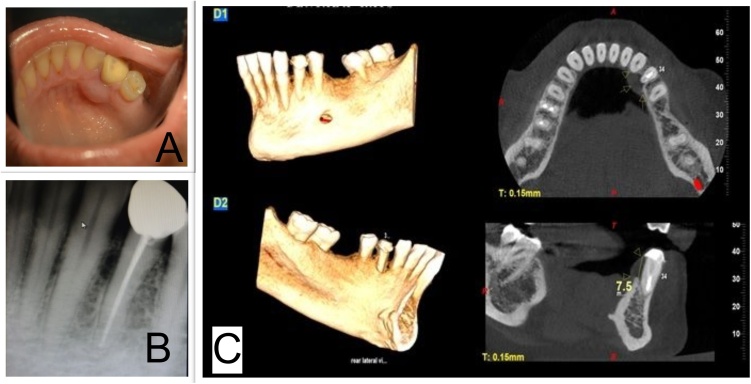
**A]** Mirror image of gingival swelling seen in lingual aspect of mandibular canine (#33) and first premolar (#34) area. **B]** IOPA X ray showing ovoid radiolucency in mesial aspect of #34. **C]** CBCT image showing the soft tissue shadow of the gingival enlargement in lingual aspect of #34 as well as the extensive bone loss in the lingual cortical bone.

Upon clinical examination, a well-circumscribed and fluctuant swelling, measuring about 10 mm in diameter, was observed at the junction of the buccal attached and free gingival margins in between the teeth #33 and #34 (Fig. 1A). The swelling was nontender and #33 was vital upon electric pulp testing. Both the teeth were not mobile. Fine needle aspiration revealed a yellow coloured fluid. Extra oral examination did not reveal any clinically discernible asymmetry, swelling or lymphadenopathy. An Intra-oral periapical (IOPA) radiograph of the site revealed a radiolucency of oval shape between the roots of teeth 33 and 34 (Fig. 1B). CBCT image showed a soft tissue shadow of a well-defined sessile growth on the lingual aspect of the cervical and middle third of 34. There was extensive loss of lingual cortical plate approximately 7.5 mm measured from the cemento-enamel junction (Fig. 1C).

After getting informed consent from the patient, scaling and root planing was done. Based on the clinical and radiographic findings, a provisional diagnosis of LPC was given. A gingivectomy procedure was done in the region. The enlargement was completely removed and curettage of the area was done. The bone defect showed expansion of the lingual cortical plate with a smoothened appearance of the bone (Fig. 3A). Sutures were placed and periodontal dressing was given. Post-op antibiotics and analgesics were prescribed. The post-operative clinical healing was uneventful (Fig. 3C & D).

**Fig. 2:**
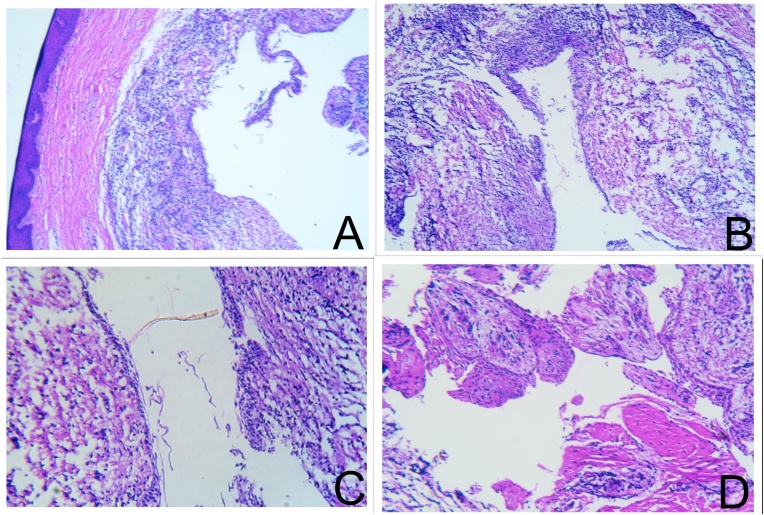
**[A-D]**: Histopathological examination of the excised lesion showing showed cystic lumen lined by 2–3 layers of non-keratinizing cuboidal cells resembling REE which were hyperplastic at places. The lining epithelium also showed clear cells, localized thickenings/plaques and mural bulges. The underlying connective stroma is dense collagenous made up of bundles of collagen fibers, fibroblasts, blood vessels and dense infiltration of chronic inflammatory cell infiltrate. The overlying epithelium is parakeratinized stratified squamous in nature and is separated from the lesional tissue by a zone of normal connective tissue stroma.

**Fig. 3:**
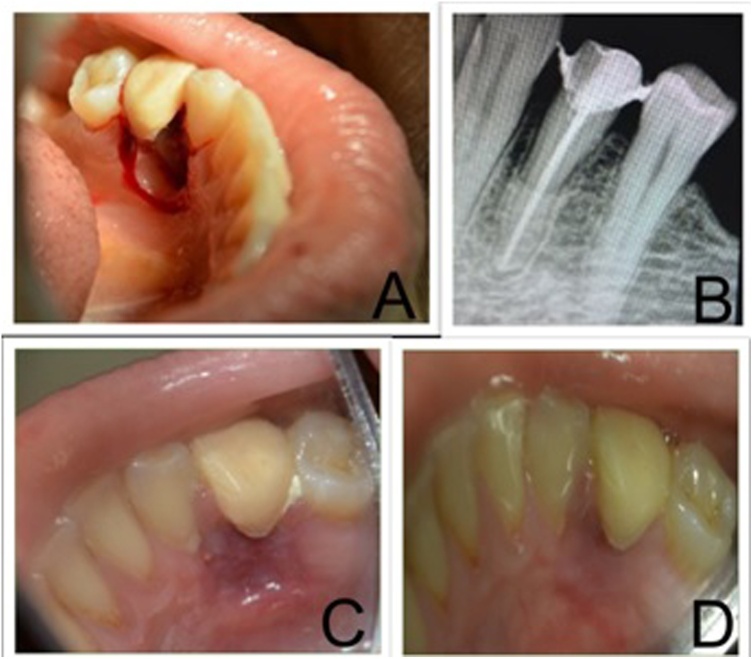
**A]** On excision of lesion, extensive destruction of bone and expansion of cortical bone seen. **B]** 4 weeks post-operative IOPA xray showing filling of bony lesion. **C]** 4 weeks post operative clinical photograph showing healing lesion (mirror image) **D]** 8 weeks post operative clinical photograph showing satisfactory healing (mirror image).

The excised soft tissue specimen was sent for histopathology examination (HPE). HPE of the excised specimen showed cystic lumen lined by 2–3 layers of non-keratinizing cuboidal cells resembling reduced enamel epithelium which were hyperplastic at places. The lining epithelium also showed clear cells, localized thickenings/plaques and mural bulges protruding into the cystic cavity. The underlying connective stroma is dense collagenous made up of bundles of collagen fibres, fibroblasts, blood vessels and dense infiltration of chronic inflammatory cell infiltrate. The overlying epithelium is parakeratinized stratified squamous in nature and is separated from the lesional tissue by a zone of normal connective tissue stroma (Fig. 2A–D). Based on the HPE and clinico-radiological correlation, a diagnosis of infected lateral periodontal cyst was established. Follow-up examinations did not reveal any clinical or radiographic evidence of recurrence of the lesion. Regeneration of bone in the defect area laterally was observed in periapical radiography at 8 weeks (Fig. 3B).

## 3 Discussion

It was 1958, that Standish and Shafer first reported LPC in literature with a series of five cases presenting in the mandible [14]. LPC is an uncommon developmental odontogenic cyst which occurs in association with vital teeth and is usually asymptomatic unless the cyst is secondarily infected. It is often reported as a by chance finding during routine radiographic evaluation. A small swelling of gingiva or alveolar mucosa has been reported occasionally [2,3]. Among all the odontogenic cysts, LPC accounts for about 0.8%–2% of all cysts. Literature shows that LPC is more prevalent in adults in the 5th–7th decade age group, without any particular preference for race or sex. The most common sites of occurrence of LPC reported include the mandibular premolar-canine region or in the maxillary anterior region [2,6,15]. In our case too, the lesion was seen in a 49 year old female patient and site was the mandibular canine-premolar region and presented as an asymptomatic swelling in the lingual gingiva.

In the etiopathogenesis of LPC, there are mainly three different views with regard to cell type of origin. Altini M and Shear M have attributed origin from reduced enamel epithelium (REE), due to the fact that the cyst is often lined by nonkeratinized epithelium similar to REE as has been shown by PCNA immunohistochemical expression [9]. Cohen D A et al., point to dental lamina remnants as cells of origin because LPC histologically shows presence of glycogen-rich clear cells which are also seen in the dental lamina [11]. Shear M pointed to epithelial cell rests of Malassez present on root surfaces playing a role in the pathogenesis of LPC [1].

LPC has a characteristic radiological presentation as a round or oval shaped well circumscribed radiolucency with a sclerotic border located between the crest and apex of alveolar process. It does not involve periodontal ligament space or cause root resorption of adjacent teeth. A similar radiographic picture may be seen in anatomic interradicular radiolucencies such as the mental foramen, nutrient canals and the maxillary sinus or in pathologies such as cysts of pulpal origin, odontomas, tumours and other cysts of jaw [16]. In 2006, Mendes RA and van der Waal have opined that more importance should be given to histological diagnosis of LPC than radiographic diagnosis [17]. In our case, the tooth (#34) was endodontically treated, with a proper apical seal. No lateral or accessory canals were seen in radiograph. The IOPA radiograph showed a typical presentation of LPC and the CBCT image showed extensive destruction of the lingual bone. The development of LPC in a non-vital tooth has not been frequently reported.

Based on the clinical and radiographic findings, a provisional diagnosis of LPC was considered and HPE was done to confirm this. LPC presents some characteristic histopathological features which include one to three cell layer thick non-keratinized epithelial lining which often resembles odontogenic epithelium or REE, which may display a palisading pattern. The epithelial lining shows areas of thickening referred to as epithelial plaques which are composed of glycogen rich clear fusiform cells. The connective tissue is rich in collagen, which may show areas of hyalinization with minimal inflammatory cell infiltrate [11,16]. In the present case, HPE showed similar findings with cystic lumen lined by of non-keratinizing cuboidal cells resembling REE, presence of clear cells, localized thickenings/plaques and mural bulges protruding into the cystic cavity. The underlying connective stroma was dense collagenous and showed dense infiltration of chronic inflammatory cell infiltrate. These findings resulted in a confirmatory diagnosis of an infected LPC.

Botryoid odontogenic cyst (BOC) also reports similar HPE findings as LPC. The differentiating feature is that BOC is seen as a multilocular radiolucency. Some authors consider BOC as a histopathologic variant of LPC, which has a greater tendency for recurrence following treatment. Altini and Shear in 1992 proposed a new classification of LPC into unicystic, polycystic or botryoid variants, all of which have a similar histopathological presentation. This classification was based on the hypothesis that all LPCs can progress to multicystic lesions [9]. Vidakovic et al. in 2016 reported that BOC develops from the cystic lining of a pre-existing LPC and presents with aggressive intrabony expansion [18]. A differential diagnosis should also include odontogenic keratocysts, pseudocysts, gingival cysts, lateral radicular cysts and other radioluscent odontogenic tumours. Careful diagnosis must be done with the help of histological examination as some of these conditions are aggressive, have high recurrence rates and have varied treatment options [8].

Literature shows that in most of the cases of LPC, the associated teeth are vital therefore extraction or endodontic treatment is not required. Treatment of LPC is conservative surgical enucleation and thorough curettage of cystic lining to remove any remnants [2,13]. Guided bone regeneration of the cystic cavity using a xenograft (Bio-Oss) and a resorbable collagen membrane (Bio-Gide) has been successfully tried in a patient with LPC [19]. In our case, we have done a gingivectomy procedure to completely remove the gingival enlargement and thoroughly curetted the region. Formosa Senande et al. have reported that after removal of lesion, bone cavity tends to get filled spontaneously [20]. Satisfactory healing of the site was observed both clinically and radiographically during post- operative evaluation. Patient has been advised periodic review for evaluation of healing and to rule out recurrence of lesion.

## 4 Conclusion

LPC is a rare developmental odontogenic cyst which should be considered in the differential diagnosis if in a radiograph cystic lesion is seen on the lateral surface of tooth especially in the mandibular canine-premolar and anterior maxillary region. It may present as an asymptomatic gingival swelling which needs to be thoroughly examined both clinically and radiologically. A histological evaluation is necessary to confirm the diagnosis. The treatment of choice is surgical removal and recurrences are infrequent.

## Declaration of Competing Interest

The authors report no declarations of interest.

## Funding

There is no funding from any source for this paper.

## Ethical approval

Written informed consent has been obtained from the patient. Her approval for publication of case details has also been received.

Since the case has been treated in a private clinic. Permission has been obtained from the clinic authorities for publication of case details.

## Consent

A fully informed written consent has been obtained from the patient.

## Author contribution

Roshni Ramesh: Conceptualization, methodology, validation, investigation, resources, writing-revision & editing & supervision.

Arun Sadasivan: Conceptualization, methodology, validation, investigation, resources, writing- original draft, visualisation & supervision.

## Registration of research studies

N/A.

## Guarantor

Dr Arun Sadasivan.

## Provenance and peer review

Not commissioned, externally peer-reviewed.
